# Standards and Guidelines for Graduate Programs in Gerontology and Geriatrics

**DOI:** 10.1093/geroni/igab046.2135

**Published:** 2021-12-17

**Authors:** Julie Masters, Rona Karasik

**Affiliations:** 1 University of Nebraska, Lincoln, Nebraska, United States; 2 Saint Cloud State University, SAINT CLOUD, Minnesota, United States

## Abstract

Graduate programs in gerontology prepare students for advanced academic and/or applied careers in aging. Programs at this level offer greater depth, breadth, and increased emphasis on theory and research. Persons completing a master’s and/or doctoral degree in gerontology or aging studies have reached the level of “gerontologist” whereby they have completed the necessary coursework in the physical, psychological, social and built environment in order to understand the unique opportunities and challenges of aging in a scholarly manner. While the depth of treatment of each topic will vary across programs, each of the AGHE Core Competencies should be mastered at a level of “analyzation and evaluation” or higher on Bloom’s Taxonomy of Educational Objectives. The current presentation addresses how the AGHE Standards and Guidelines for graduate programs in gerontology were updated as a competency-based curriculum that includes coursework, practicum, and a culminating project (e.g., thesis, comprehensive exam, and/or dissertation).

